# A new split‐luciferase complementation assay identifies pentachlorophenol as an inhibitor of apoptosome formation

**DOI:** 10.1002/2211-5463.12646

**Published:** 2019-05-29

**Authors:** Amin Tashakor, Mahshid H‐Dehkordi, Enda O'Connell, Sergi Gomez Ganau, Rafael Gozalbes, Leif A. Eriksson, Saman Hosseinkhani, Howard O. Fearnhead

**Affiliations:** ^1^ Pharmacology and Therapeutics School of Medicine NUI Galway Ireland; ^2^ Genomics and Screening Core National Centre for Biomedical Engineering Science NUI Galway Ireland; ^3^ PROTOQSAR Valencia Spain; ^4^ University of Gothenburg Sweden; ^5^ Tarbiat Modares University Tehran Iran

**Keywords:** Apaf‐1, apoptosome, pentachlorophenol, reproductive toxicity, screening, split luciferase complementation assay

## Abstract

The expense and time required for *in vivo* reproductive and developmental toxicity studies have driven the development of *in vitro* alternatives. Here, we used a new *in vitro* split luciferase‐based assay to screen a library of 177 toxicants for inhibitors of apoptosome formation. The apoptosome contains seven Apoptotic Protease‐Activating Factor‐1 (Apaf‐1) molecules and induces cell death by activating caspase‐9. Apaf‐1‐dependent caspase activation also plays an important role in CNS development and spermatogenesis. In the *in vitro* assay, Apaf‐1 fused to an N‐terminal fragment of luciferase binds to Apaf‐1 fused to a C‐terminal fragment of luciferase and reconstitutes luciferase activity. Our assay indicated that pentachlorophenol (PCP) inhibits apoptosome formation, and further investigation revealed that PCP binds to cytochrome *c*. PCP is a wood preservative that reduces male fertility by ill‐defined mechanisms. Although the data show that PCP inhibited apoptosome formation, the concentration required suggests that other mechanisms may be more important for PCP's effects on spermatogenesis. Nonetheless, the data demonstrate the utility of the new assay in identifying apoptosome inhibitors, and we suggest that the assay may be useful in screening for reproductive and developmental toxicants.

**Abbreviations**
ATP: adenosine triphosphate
dATP: 2′‐deoxyadenosine triphosphate
DEVD‐AMC: *N*‐acetyl‐Asp‐Glu‐Val‐Asp‐7‐amido‐4‐methylcoumarin
DMEM: Dulbecco's modified Eagle's medium
FBS: fetal bovine serum
HEPES: 4‐(2‐hydroxyethyl)‐1‐piperazineethanesulfonic acid
NS3694: 4‐chloro‐2‐[[3‐(trifluoromethyl)phenyl]carbamoylamino]benzoic acid
PCP: pentachlorophenol

*In vivo* reproductive and developmental toxicity studies require the most time and cost the most money when assessing the toxicity of a chemical 1. This cost is driving the development of alternatives to *in vivo* testing and even a revolution in how we think about reproductive and developmental toxicity studies 2. Information about how developmental and reproductive processes are poisoned by chemicals can be used to define adverse outcome pathways (AOPs) 3. These pathways can be used to identify key molecular events that can act as surrogates for toxicity and be the basis of new *in vitro* tests. The AOP approach is limited as it is built upon our existing knowledge of toxicants. In principle, the chemical‐centric AOP approach could be complemented by using existing data from knockout studies of development and reproduction. In this case, the key molecular events are used to build new *in vitro* toxicity assays, even if no chemical has yet been shown to act by this mechanism. This strategy broadens the type of assay being used and improves the likelihood of detecting toxicants with unexpected mechanisms of action.

Apoptotic Protease‐Activating Factor‐1 (Apaf‐1) is a 140 kDa cytosolic protein identified by its ability to activate caspase‐9, a key caspase in the mitochondrial apoptotic pathway 4. In the mitochondrial pathway, the release of cytochrome *c* from mitochondria allows it to bind to Apaf‐1, causing 2′‐deoxyadenosine triphosphate (dATP)/adenosine triphosphate (ATP)‐dependent Apaf‐1 oligomerization into a heptameric complex called the apoptosome. Caspase‐9 is activated when it binds the apoptosome, and active caspase‐9 in turn activates caspase‐3 activation in a cascade of events that ultimately kills a cell by apoptosis.

Apoptotic Protease‐Activating Factor‐1 null mice typically die shortly after birth and show exencephaly, cranioschisis, and forebrain hyperplasia 5, 6. A similar phenotype is seen in transgenic mice lacking caspase‐9 7, 8, supporting the idea that these effects are mediated through the mitochondrial pathway. A small proportion of Apaf‐1 null mice survive to adulthood, but the males are infertile due to a failure of spermatogenesis 9. A similar phenotype is seen in flies lacking DARK and DRONC, the fly Apaf‐1 and caspase‐9, indicating a conserved role for Apaf‐1 in this process 10, 11.

Using a new *in vitro* assay based on split luciferase complementary assay 12, we screened a panel of 177 toxicants to identify modulators of apoptosome formation. We show data validating the assay as a measure of apoptosome formation and report that pentachlorophenol (PCP) prevents apoptosome formation, most likely by binding to cytochrome *c*. PCP is a wood preservative with a range of toxicities, including reproductive toxicity. Although the inhibition of apoptosome provides a mechanistic explanation for how PCP reduces male fertility, the high concentration of PCP required for apoptosome inhibition suggests that other mechanisms explain the reproductive toxicity. Nonetheless, the assay is suitable for a larger scale screening activity designed to identify chemicals that affect Apaf‐1 function and should therefore be further investigated as potential reproductive or developmental toxicants.

## Results

### Assay validation

**Figure 1:**
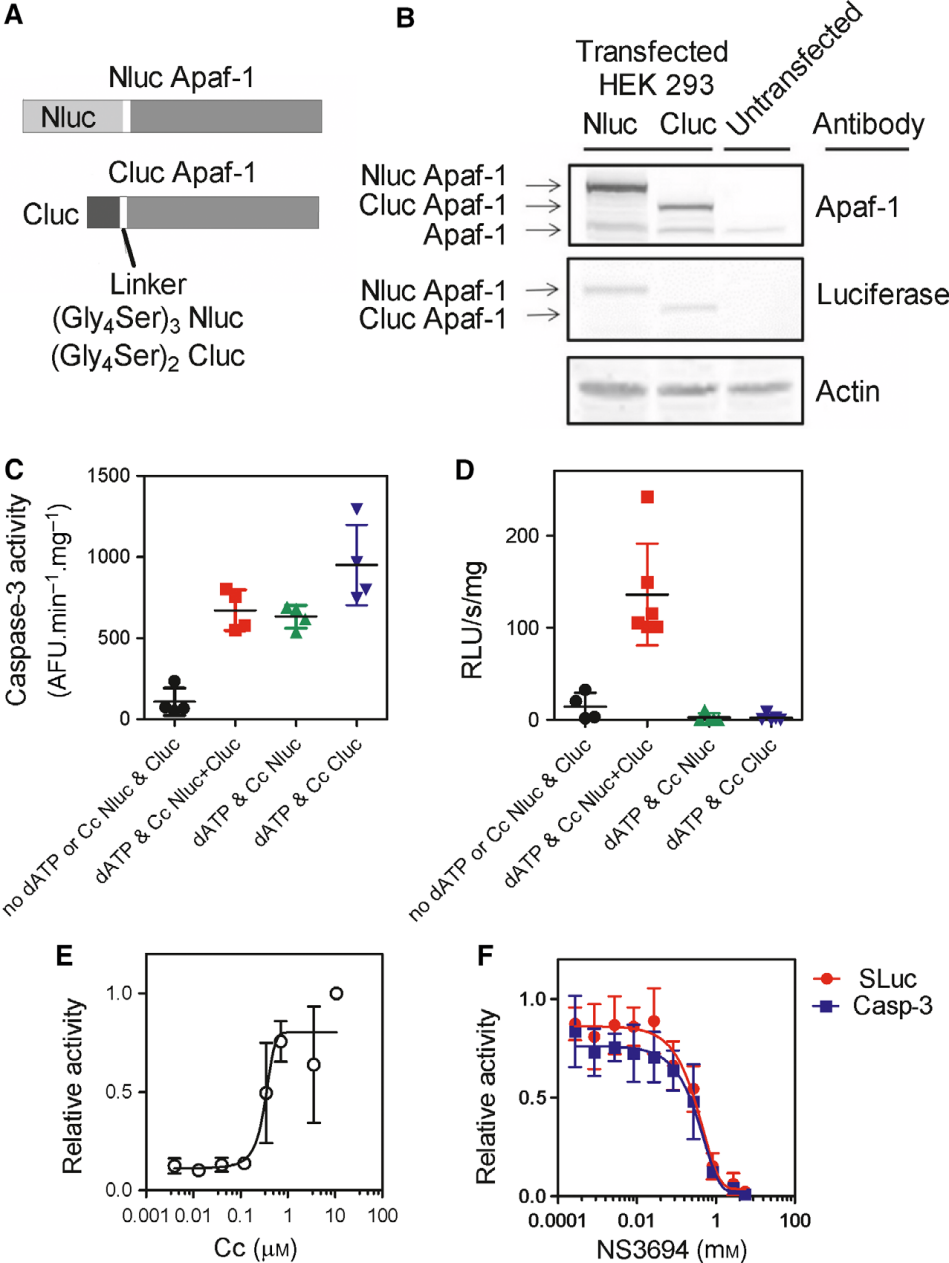
(A) Schematic representation of the fusion protein. N‐terminal (residues 1‐416) and C‐terminal (residues 394‐550) fragments of firefly luciferase fused to Apaf‐1 by insertion of a flexible linker, made of Gly and Ser residues. (B) S‐100 extract prepared from cells transiently transfected with Nluc‐Apaf1 or Cluc‐Apaf1. Cells were harvested 24 h after transfection, and expression of recombinant proteins was assessed by immunoblotting. (C) Caspase‐3‐like activity of the S‐100 extract was measured using DEVD‐AMC in the presence and absence of dATP/Cc. (D) Extracts (1.6 mg·mL^−1^) expressing Nluc/Apaf1 or Cluc/Apaf1 were mixed 1 : 1 in the presence and absence of 1.6 μm cytochrome *c* and 1 mm dATP, and split luciferase activity was measured. (E) The effect of cytochrome *c* concentration on split luciferase activity was assessed. (F) The effect of NS3694 on inhibition of split luciferase activity (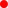) and caspase‐3‐like activity (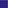). Data are the mean ± SD of three independent experiments.

A split luciferase assay has been reported earlier for detecting the apoptosome in cells 13. However, the cytochrome *c* dependence of the assay was not tested nor was the size of the Apaf‐1 complex determined. To further investigate the usefulness of this assay, we made split luciferase Apaf‐1 constructs (Fig. 1A) and transiently overexpressed the constructs in human embryonic kidney (HEK) 293 cells. The overexpression of proteins was detected using an anti‐Apaf‐1 antibody and an anti‐luciferase antibody. Figure 1B shows detection of the expressed proteins at the predicted molecular mass.

We prepared S‐100 extracts 14 from untransfected or transfected HEK 293 cells expressing either Nluc/Apaf‐1 or Cluc/Apaf‐1. Extracts had low caspase‐3 activity, but addition of cytochrome *c* and dATP (dATP/Cc) significantly induced caspase‐like activity as assessed by cleavage of *N*‐acetyl‐Asp‐Glu‐Val‐Asp‐7‐amido‐4‐methylcoumarin (DEVD‐AMC) (Fig. 1C). These data show that the competent apoptotic machinery was extracted from the cells.

We then measured the luciferase activity in the extracts. Addition of dATP/Cc did not increase luciferase activity in extracts from Nluc Apaf‐1‐expressing cells or in extracts from Cluc Apaf‐1‐expressing cells, which is expected as only half the luciferase enzyme is present in each reaction (Fig. 1D). However, when Nluc/Apaf‐1 and Cluc/Apaf‐1 extracts were mixed, addition of dATP/Cc increased luciferase activity significantly. Even in the absence of added dATP/Cc, there was more luciferase activity than was detected in the Nluc‐alone or Cluc‐alone controls (Fig. 1D). This luciferase activity represents either Apaf‐1‐Apaf‐1 interactions triggered by high Apaf‐1 concentrations or apoptosome formation induced by endogenous dATP/ATP and cytochrome *c* that is already present in the cell extract.

To test this, extracts were incubated with S‐Sepharose to deplete cytochrome *c* without removing Apaf‐1, caspase‐9, or caspase‐3 14. After this manipulation, extracts from untransfected cells showed very low caspase‐3 activity whereas high caspase activity was detected in the extracts from cells overexpressing Apaf‐1 (Fig. S1). Addition of exogenous dATP/Cc increased caspase‐3 activity in both cases. These data suggest that overexpression of Apaf‐1 increased apoptosome formation, but that after extract preparation, further apoptosome could be triggered by addition of dATP/Cc.

To test whether the concentration of cytochrome *c* required for luciferase activity was similar to that of apoptosome formation, cytochrome *c* was titrated into extracts containing both Nluc/Apaf‐1 and Cluc/Apaf‐1. Maximal luciferase activity was achieved at ~ 0.78 μm cytochrome *c*, and the EC_50_ was estimated at 0.15 μm (Fig. 1E). These data are in good agreement with previous reports 15, 16.

We reasoned that 4‐chloro‐2‐[[3‐(trifluoromethyl)phenyl]carbamoylamino]benzoic acid (NS3694), a diaryl urea that has been reported to block apoptosome formation 17, should prevent reconstitution of luciferase activity. We therefore added NS3694 to extracts and tested whether luciferase activity was reconstituted. Addition of NS3694 to diluted extracts containing both Nluc and Cluc/Apaf‐1 blocked the increase in luciferase activity induced by dATP/Cc (Fig. 1F). Maximal inhibition was achieved at ~ 2 mm with an estimated IC_50_ of 170 μm. We also tested the inhibition of caspase activation and found good agreement between the two assays, which is expected if the assay is measuring apoptosome formation.

The apoptosome complex has a *M*_r_ of ~ 700 kDa. Endogenous Apaf‐1 could generate the caspase activity observed, while luciferase activity requires only dimerization of the Apaf‐1 fusion proteins. Therefore, it was possible that the signal we detected was not from an apoptosome *per se*, but from Apaf‐1 fusion proteins forming a different type of complex.

**Figure 2:**
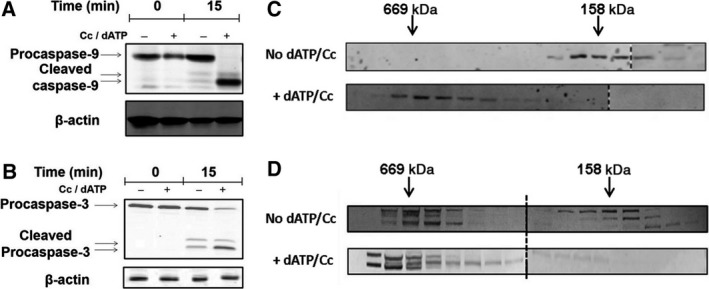
(A) HEK 293 S‐100 extract was incubated for 15 min in the presence and absence of dATP/Cc, and the processing of caspase‐9 and (B) caspase‐3 was detected by immunoblotting. (C) To detect the apoptosome formation, 1 mL HEK 293 S‐100 extract was incubated for 15 min in the presence and absence of dATP/Cc and loaded on Sephacryl 300 HR column. The fractions were collected, concentrated, and immunoblotted to detect Apaf‐1. (D) 1 mL of Nluc‐Apaf1 and Cluc‐Apaf1 extracts was mixed 1 : 1, incubated for 15 min in the presence and absence of dATP/Cc, and loaded on a Sephacryl 300 HR column. The fractions were collected, concentrated, and immunoblotted to detect Apaf‐1.

To test this possibility, gel filtration chromatography was performed to determine the apparent molecular size of protein complexes containing Nluc and Cluc/Apaf‐1. S‐100 extract was incubated for 15 min and processing of procaspase‐9 and procaspase‐3 detected by immunoblotting (Fig. 2A,B). As expected, dATP/Cc‐dependent caspase activation was observed. Gel filtration chromatography showed that the formation of a ~ 700 kDa Apaf‐1 complex was induced by dATP/Cc (Fig. 2C). When proteins from a mixture of Nluc and Cluc/Apaf‐1 extracts were resolved by gel filtration, Apaf‐1 was detected as both monomeric protein and a complex of ~ 700 kDa, even in the absence of dATP/Cc (Fig. 2D). However, addition of dATP/Cc increased the proportion of Apaf‐1 associated with the ~ 700 kDa complex and the monomeric form became undetectable (Fig. 2D). These data are consistent with Fig. S1 and suggest that overexpression of Apaf‐1 increased complex formation before the addition of dATP/Cc, either because of the high Apaf‐1 concentration or because of the endogenous dATP/Cc, but that addition of dATP/Cc induced further complex formation.

### Screening a toxicant library

**Figure 3:**
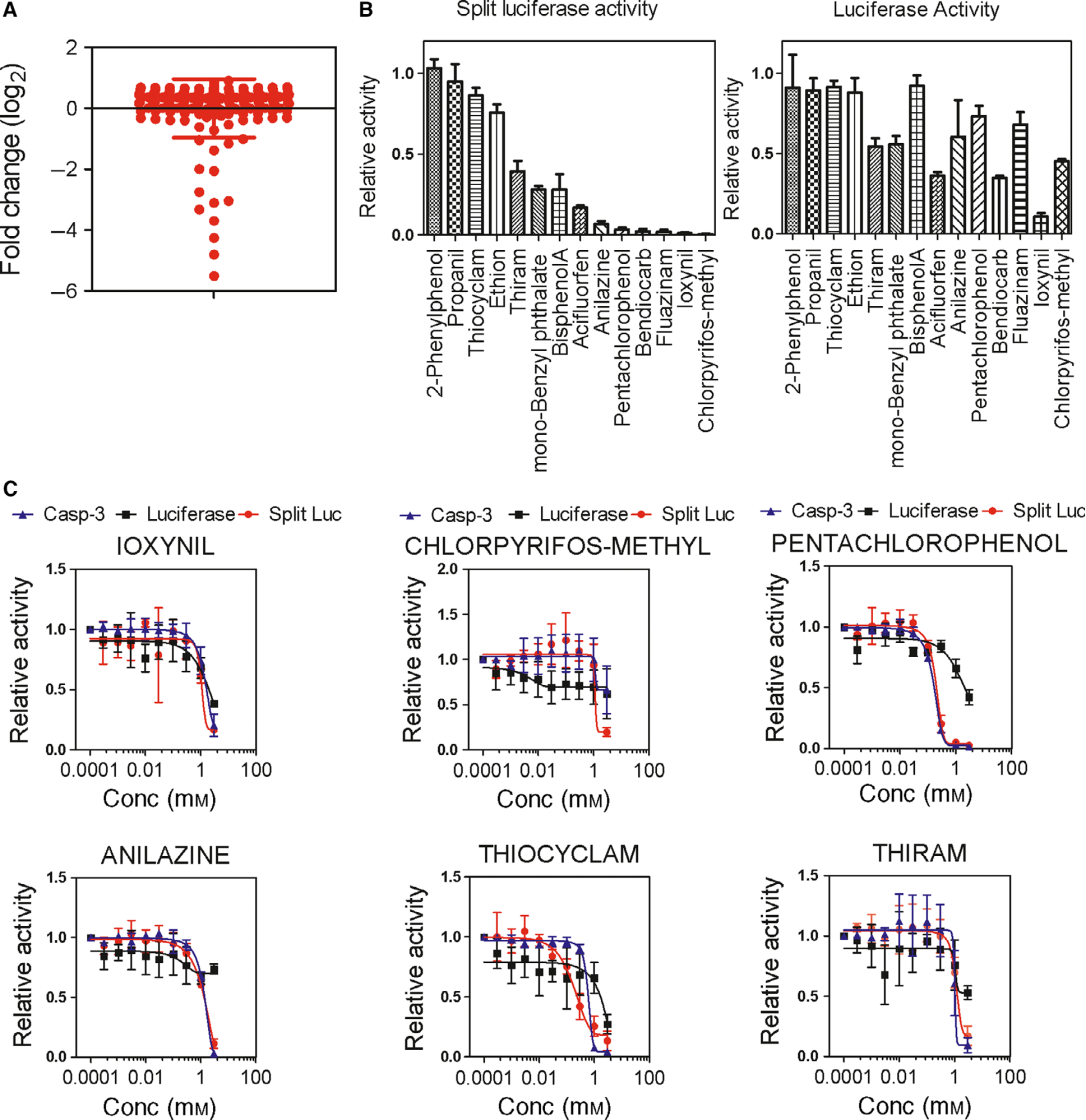
Screening and analysis of the toxicant library. (A) Screening of 177 toxicants showing log_2_ fold change relative to untreated control. (B) Fourteen compounds were defined as potential hits and re‐assessed by comparing inhibition of split luciferase activity (left‐hand panel) and luciferase activity (right‐hand panel). (C) The concentration‐dependent effect of six compounds on luciferase activity (■), split luciferase activity (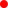), and caspase‐3‐like activity (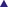) was assessed. Data are the mean ± SD of three independent experiments.

We next wanted to test whether the assay could work in a multiwell plate format. We therefore tested a library of 177 persistent organic pollutants for the ability to inhibit apoptosome formation. Toxicants were initially screened at a concentration of 1 mm with NS3694 used as a positive control (Fig. 3A). As expected, the screen identified NS3694, and interestingly, 14 toxicants showed some inhibition of apoptosome formation, albeit at a high concentration. These 14 toxicants with inhibitory activity were then tested at 1 mm against an extract from cells expressing *Photinus pyralis* firefly luciferase to identify those toxicants that act directly on the luciferase enzyme rather than on apoptosome formation (Fig. 3B). Six toxicants that showed the greatest specificity for split luciferase activity were then titrated to determine IC_50_ values in different assays (split luciferase activity, *P. pyralis* firefly luciferase activity, and caspase‐3‐like activity; Fig. 3 and Table 1). The concentration responses detected for ioxynil in all three assays were similar, suggesting that ioxynil's effects were not specific for the apoptosome (Fig. 3C). Thiram, thiocyclam, chlorpyrifos, and anilazine all inhibited split luciferase activity, caspase‐3 activity, and luciferase activity. A difference between the effect on the split luciferase activity and the luciferase activity was only seen at the highest concentration tested (Fig. 3C). However, PCP showed a clear difference between its effect on the split luciferase and caspase activity and its effect on the luciferase assay (Fig. 3C). PCP also had the lowest IC_50_ in the split luciferase assay. We therefore concentrated on PCP.

**Table 1:** IC_50_ values calculated for luciferase (Luc), split luciferase (S. Luc), and caspase‐3 (Casp‐3) activity

Compound	IC_50_ (mm)			Structure
	Luc	S. luc	Casp‐3	
Chlorpyrifos‐methyl	> 3	> 3	> 3	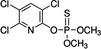
Anilazine	> 3	2.99	2.83	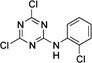
Ioxynil	> 3	1.18	> 3	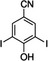
Thiocyclam	> 3	0.20	0.51	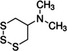
Pentachlorophenol	> 3	0.17	0.15	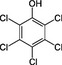
Thiram	> 3	1.38	1.05	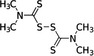

### PCP prevents the formation of the apoptosome

**Figure 4:**
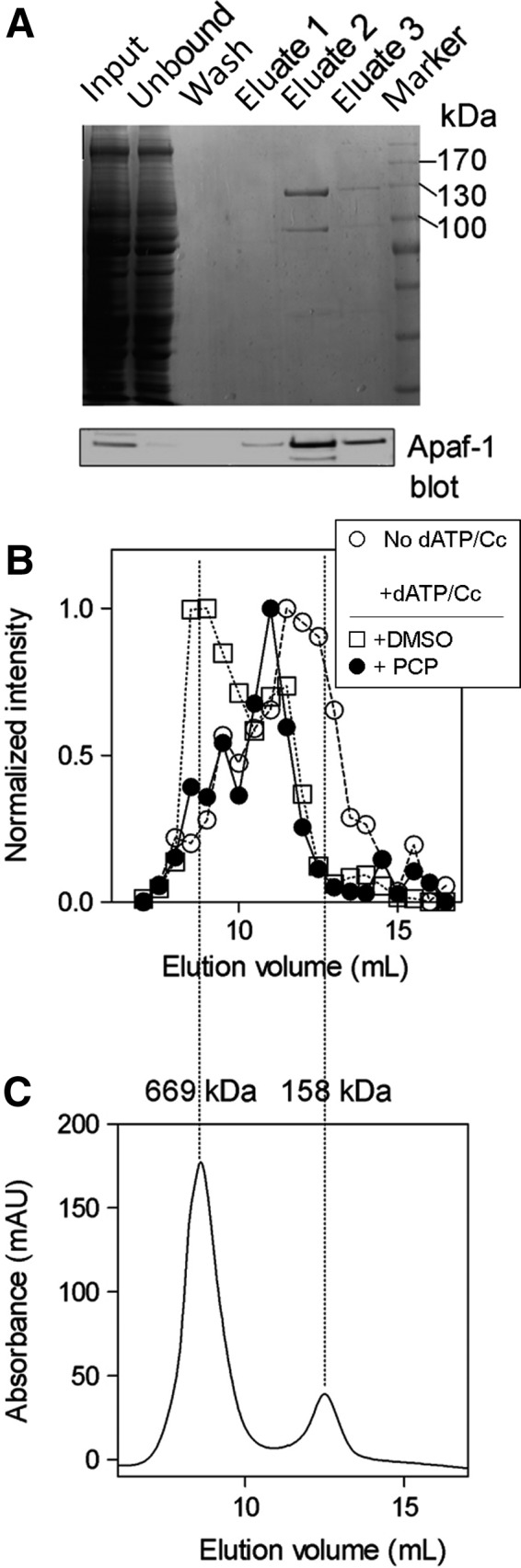
Direct effect of PCP on apoptosome formation. (A) Purified recombinant Apaf‐1 (rApaf‐1). Human Apaf‐1 was expressed in Sf21 cells and purified via His‐tag affinity chromatography. The fractions were loaded on SDS/PAGE, and the rApaf‐1 was probed using anti‐Apaf1 antibody. (B) PCP prevents rApaf‐1 oligomerization. Gel filtration was performed, and the fractions were collected and analyzed by dot blot using an anti‐Apaf‐1 antibody. The intensity of the dots was then quantified (○) no dATP/Cc; (□) dATP/Cc; (●) dATP/Cc + 1 mm PCP. (C) Thyroglobulin (669 kDa) and aldolase (158 kDa) were used as protein markers.

Pentachlorophenol inhibited apoptosome formation in a whole extract that contains many proteins. In order to test whether PCP directly targets the proteins involved in apoptosome, we used purified recombinant Apaf‐1 (Fig. 4A), cytochrome *c*, and dATP and assessed apoptosome formation in the presence and absence of PCP by gel filtration. Dot blot analysis using an anti‐Apaf1 antibody showed that the addition of dATP/Cc induced rApaf‐1 oligomerization into a complex of ~ 700 kDa fraction and that addition of PCP prevented rApaf‐1 oligomerization (Fig. 4B and 4C).

Oligomerization to form the apoptosome also requires Apaf‐1 to bind ATP or dATP. PCP inhibits a kinase by preventing ATP binding 18. It was therefore possible that PCP blocked apoptosome formation by preventing dATP/ATP binding. While our data showed that PCP acts directly on apoptosome formation, we could not tell whether PCP bound Apaf‐1 or cytochrome *c*.

**Figure 5:**
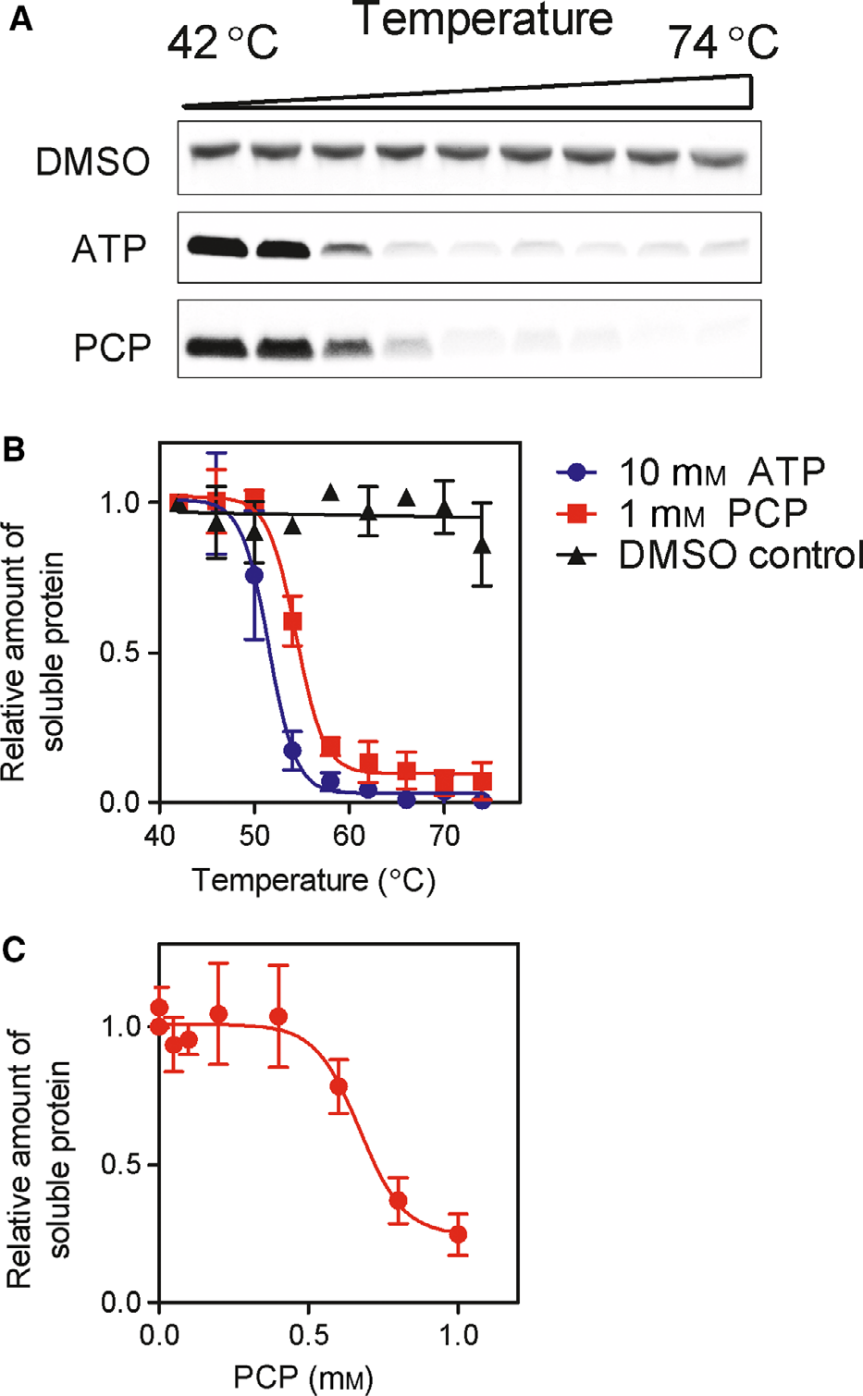
PCP affects cytochrome *c*. Cytochrome *c* was incubated at increasing temperatures (42, 46, 50, 54, 58, 62, 66, 70, and 74 °C) with PCP or without (DMSO). Cytochrome *c* was also incubated with ATP (10 mm). (A) The amount of cytochrome *c* remaining soluble was assessed by Coomassie Blue. (B) The data from three experiments were quantified (DMSO: ▲; PCP: 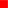; ATP: 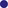; data are mean ± SD). (C) Cytochrome *c* was incubated at 58 °C with different concentrations of PCP and the amount of cytochrome *c* remaining soluble assessed by Coomassie Blue staining and quantified. The data are from three experiments (mean ± SD).

To determine which protein was the target, a thermal stability shift assay (TSA) was performed 19. Cytochrome *c* was very stable at high temperatures (74 °C; Fig. 5A). However, incubation with 1 mm PCP reduced the thermostability of cytochrome *c*, suggesting a direct interaction between them (Fig. 5B). ATP (10 mm) binds to cytochrome *c* and inhibits apoptosome formation 20 and was used as a positive control (Fig. 5B). A thermostability shift assay using rApaf‐1 did not show any changes in the stability of rApaf‐1 in the presence of PCP (Fig. S2). These data suggest that cytochrome *c* and not Apaf‐1 is the target for PCP. To further test this idea, we investigated the concentration dependence of PCP's effects. These experiments showed that the EC_50_ for cytochrome c destabilization was ~ 600 μm, or approximately four times that of the IC_50_ for inhibition of the apoptosome (Fig. 5C).

## Discussion

Knockout studies show Apaf‐1 is a key protein in important cellular processes and that its dysfunction is linked to different pathologies including defects in central nervous system development and spermatogenesis 5, 6, 9. Chemicals that block Apaf‐1 function are therefore predicted to be potential developmental and reproductive toxicants. Screening large numbers of chemicals for the ability to inhibit Apaf‐1 is impractical using existing assays, which are time‐consuming and suited to only a small number of samples. In the current study, we established an assay based on split luciferase reporters to investigate the homotypic Apaf‐1 interactions *in vitro* with the aim of using this new tool to study apoptosome complex formation and identify Apaf‐1 activators and inhibitors. The assay reported here shows significant advantages over other assays used to detect Apaf‐1 modulators as Apaf‐1 oligomerization can be quickly measured in multiple samples. The assay was validated by assessing key criteria: dependence on dATP/Cc; sensitivity to a known apoptosome inhibitor; and the formation of an appropriately sized oligomer of ~ 700 kDa.

The assay was used to screen a library composed of 177 toxic compounds. PCP is mainly used for maintenance and preservation of wood products. PCP showed the lowest IC_50_ value (~ 170 μm) for both caspase and luciferase activity of all the toxicants tested here. Epidemiological studies report that the general population have low‐level contamination (< 70 ppb) that is far below the levels that inhibit apoptosome formation. However, occupational exposures include plasma levels as high as 0.319 mm 21, or approximately twice PCP's IC_50_ in the apoptosome assay. In addition, animal studies show that PCP causes testicular atrophy and reduces spermatid numbers and male fertility 22. The importance of Apaf‐1 in spermatogenesis, together with the data presented here, suggests that inhibition of Apaf‐1 may contribute to PCP's reproductive toxicity.

However, PCP also acts to uncouple respiration. Other uncouplers such as 2,4‐dinitrophenol, dinoseb, and 4,6‐dinitro‐o‐cresol also affect sperm viability at concentrations of ~ 10^−5^–10^−4^ m. The severity of the effect on sperm correlates with chemical's ability to uncouple respiration 23 and the IC_50_ for PCP uncoupling respiration is ~ 5 × 10^−6^ m 24, a concentration approximately 30‐fold lower than that required to inhibit apoptosome formation. Thus, while PCP inhibits apoptosome formation, this probably makes only a minor, if any, contribution to the reproductive toxicity of PCP *in vivo*.

In conclusion, we validated a new assay to detect inhibitors of the apoptosome and demonstrated it can be used to screen a small chemical library of toxicants. We suggest the assay could be used to screen large chemical collections and that the apoptosome inhibitors that are identified are potentially reproductive or developmental toxicants. This assay could therefore be a useful addition to the battery of *in vitro* reproductive and developmental toxicity tests.

## Materials and methods

### Plasmid construction

Apoptotic Protease‐Activating Factor‐1 XL was PCR‐amplified using high‐fidelity PrimeSTAR GXL DNA Polymerase (Clontech, Mountain View, CA, USA) from an Apaf‐1 FastBac vector 25. N‐terminal (1‐416) and C‐terminal (395‐550) luciferase fragments were PCR‐amplified from pGL3 vector encoding *P. pyralis* luciferase 26 and fused to Apaf‐1 using in‐fusion cloning system. A flexible Gly and Ser linker joined the luciferase fragments to Apaf‐1 cDNA. The sequencing of the cloned fragments was confirmed by primer walking sequencing.

### Cell culture, transfection, and cell extract preparation

HEK 293 cells were cultured in DMEM supplemented with FBS (10%v/v). 4 × 10^8^ HEK 293 cells were seeded in 15 cm diameter dishes a day before transfection. Polyethylenimine‐based transfection was carried out by mixing 55 μg plasmid with 250 μL of polyethylenimine (1 mg·mL^−1^) for each 15 cm diameter dish. Cells were incubated for 24 h at 37 °C, 5% CO_2_. S‐100 extract was made as previously described 27. Briefly, cells were harvested by trypsinization and resuspended in extraction buffer [50 mm 4‐(2‐hydroxyethyl)‐1‐piperazineethanesulfonic acid (HEPES) pH 7.4, 10 mm KCl, 5 mm ethylene glycol‐bis(b‐aminoethyl ether)‐N,N,N0,N0‐tetraacetic acid, 2 mm MgCl_2_, 1 mm DTT plus cytochalasin B 10 μg·mL^−1^, PMSF 100 μm, and protease inhibitor cocktail]. Cells were lysed by three freeze–thaw cycles in liquid nitrogen along with vigorous mixing and the lysate centrifuged for 1 h at 100 000 ***g***. Protein concentration was determined by Bradford assay. Cell extract was aliquoted into fractions, snap‐frozen, and stored at −80 °C until use.

### Caspase activity

Caspase activity was measured using DEVD‐AMC as described 28. Briefly, 15 μL of extract was mixed with 185 μL of assay buffer containing HEPES 20 mm, 40 μm DEVD‐AMC, and 1 mm DTT in the presence and absence of cytochrome *c* (1.6 μm) and dATP (1 mm). Fluorescence intensity was normalized to the protein concentration.

### Luciferase assay

Ice‐cold extracts from N‐luc‐ and C‐luc‐expressing cells (5 μL of each) were mixed in a well of a 96‐well plate. Then, the following reagents were added in order: cytochrome *c*, dATP (to 1.6 μm and 1 mm final concentration, respectively), and 30 μL of One‐Glo Luciferase Assay System. The generation of light was then detected immediately using a VICTOR Plate Reader at 25 °C over 15 min.

### Immunoblotting

Sixty microgram of the cell extract was loaded onto an 8% polyacrylamide gel, separated by electrophoresis, and subsequently transferred onto a nitrocellulose membrane. The membrane was blocked (PBS 8% skimmed milk) at room temperature for 2 h and then incubated at 4 °C overnight with primary anti‐Apaf1 monoclonal antibody (AdipoGen, San Diego, CA, USA; AG‐20T‐0134‐c100), anti‐luciferase (Abcam, Cambridge, UK; ab21176), anti‐caspase‐9 (Cell Signaling Technology, Danvers, MA, USA; 9502), anti‐caspase‐3 (Cell Signaling Technology; 9662), cytochrome *c* (BD Pharmingen, San Diego, CA, USA; 556433), or actin (Proteintech, Rosemont, IL, USA; 66009‐1‐Ig), all diluted 1 : 1000. The membrane was washed with PBS containing 0.05% Tween‐20 (PBST) before being incubated with secondary antibody diluted 1 : 10 000: goat anti‐rat (Li‐COR, 925‐32219), goat anti‐rabbit (Li‐COR, 926‐32211), and goat anti‐mouse (Li‐COR, 926‐68020) in PBST. The membrane was then scanned using a LI‐COR system.

### Screening

A library of known human toxicants (Table S1), including toxicants known to interfere with spermatogenesis, was tested to identify those that reduce luciferase activity in the split luciferase assay. Toxicants were screened at 1 mm. Screening was performed using the PerkinElmer JANUS Automated Workstation in 96‐well plates. Briefly, 10 μL of Nluc/Apaf‐1 and Cluc/Apaf‐1 extract was added and mixed with 5 μL of 1 mm toxicant library compounds and incubated for 10 min at 4 °C. Then, 7 μL of dATP/Cc (1 mm and 1.6 μm final concentration, respectively) was added and mixed. Split luciferase activity was measured by adding 33 μL of One‐Glo Luciferase Assay System (Promega, Madison, WI, USA) to each well and luminescence signal recorded over 15 min. An inhibitor was defined as any toxicant that reduced mean split luciferase activity by more than Q1 minus 1.7× interquartile range.

### Production of recombinant Apaf‐1 using Baculovirus Expression System

The cDNA encoding His‐tag Apaf‐1 was cloned in pFastBac donor vector and transformed into DH10Bac *Escherichia coli* competent cells. The recombinant bacmids were then extracted after antibiotic selection and blue‐white screening and confirmed by partial sequencing. The DNA was used for transfecting Sf21 insect cells using Cellfectin Reagent. The expression of proteins was analyzed 3 days after transfection by immunoblotting. The viral stock was amplified to 70 mL and used for infection of 500 mL Sf21 cell culture at a density of 2.2 × 10^6^ cells per mL. The cells were harvested 37 h postinfection. The cell pellet was resuspended in five volumes of a buffer containing 20 mm HEPES pH 8.5, 10 mm KCl, 1.5 mm MgCl_2_, 1 mm DTT, 0.1 mm PMSF, and protease inhibitor cocktail and lysed by homogenization. S‐100 extract was prepared and loaded on 3.5‐mL nickel affinity column. The column was washed using 10 volumes of washing buffer A (20 mm HEPES pH 8.5, 5 mm β‐mercaptoethanol, 500 mm KCl, 20 mm imidazole, and 10% glycerol), two volumes of washing buffer B (20 mm HEPES pH 8.5, 5 mm β‐mercaptoethanol, 1 m KCl, and 10% glycerol), and two volumes of washing buffer A. The column was eluted with 20 mm HEPES pH 8.5, 100 mm KCl, 250 mm imidazole, 5 mm β‐mercaptoethanol, and 10% glycerol. Buffer exchange was then performed in 20 mm HEPES pH 8.5, 10 mm KCl, 1 mm DTT, and 10% glycerol using 100 molecular weight cutoff protein concentrators (Thermo Fisher, Dublin, Ireland; 88503). The proteins were then aliquoted and stored at −80 °C.

### Thermal stability shift assay

Fifty microliters of cytochrome *c* (1 mg·mL^−1^) was incubated alone or in the presence of 10 mm ATP or 1 mm PCP and heated for 7 min at a range of temperatures (42–74 °C) in a Veriti 96‐well Thermal Cycler (Applied Biosystem, Foster City, CA, USA). The reactions were then centrifuged at 16,000 x *g* for 30 min to pellet insoluble protein. 60 ng of rApaf‐1 was also used in the TSA in the same way. 10 μL of the supernatant was then used for SDS/PAGE. Immunoblotting was performed to detect the proteins.

## Data analysis

Data were analyzed using graphpad prism (San Diego, CA, USA).

## Conflict of interest

The authors declare no conflict of interest.

## Author contributions

AT contributed to cloning and cell‐free system experiments. MH constructed the library. SH conceived the assay. EOC provided screening support. HF designed the experiment, analyzed and interpreted the data, conceived the assay, wrote the manuscript, and provided funding.

## Supporting information


**Fig. S1.** Depletion of cytochrome *c* from cell extracts. S‐100 extracts were incubated with S‐sepharose, which binds cytochrome *c* but not Apaf‐1, caspase‐9 or caspase‐3. Caspase‐3 activity was then assessed, either with or without addition of exogenous dATP/Cc.

**Fig. S2.** Thermal stability assay with Apaf‐1. Recombinant human Apaf‐1 (rApaf‐1) was expressed in Sf21 cells and purified via His‐tag affinity chromatography (see Fig. 4). Purified rApaf‐1 was then incubated with PCP (1 mm) or without (DMSO) at different temperatures to denature the protein. Denatured protein was removed by centrifugation and the amount of native rApaf‐1 was assessed by immunoblot.

**Table S1**. toxicant and screen details
