# Supplementary figures and images for: A new split‐luciferase complementation assay identifies pentachlorophenol as an inhibitor of apoptosome formation
Source: FEBS Open Bio. 2019 May 29;9(7):1194–203. doi: 10.1002/2211-5463.12646 (PMC6609562; doi:10.1002/2211-5463.12646)

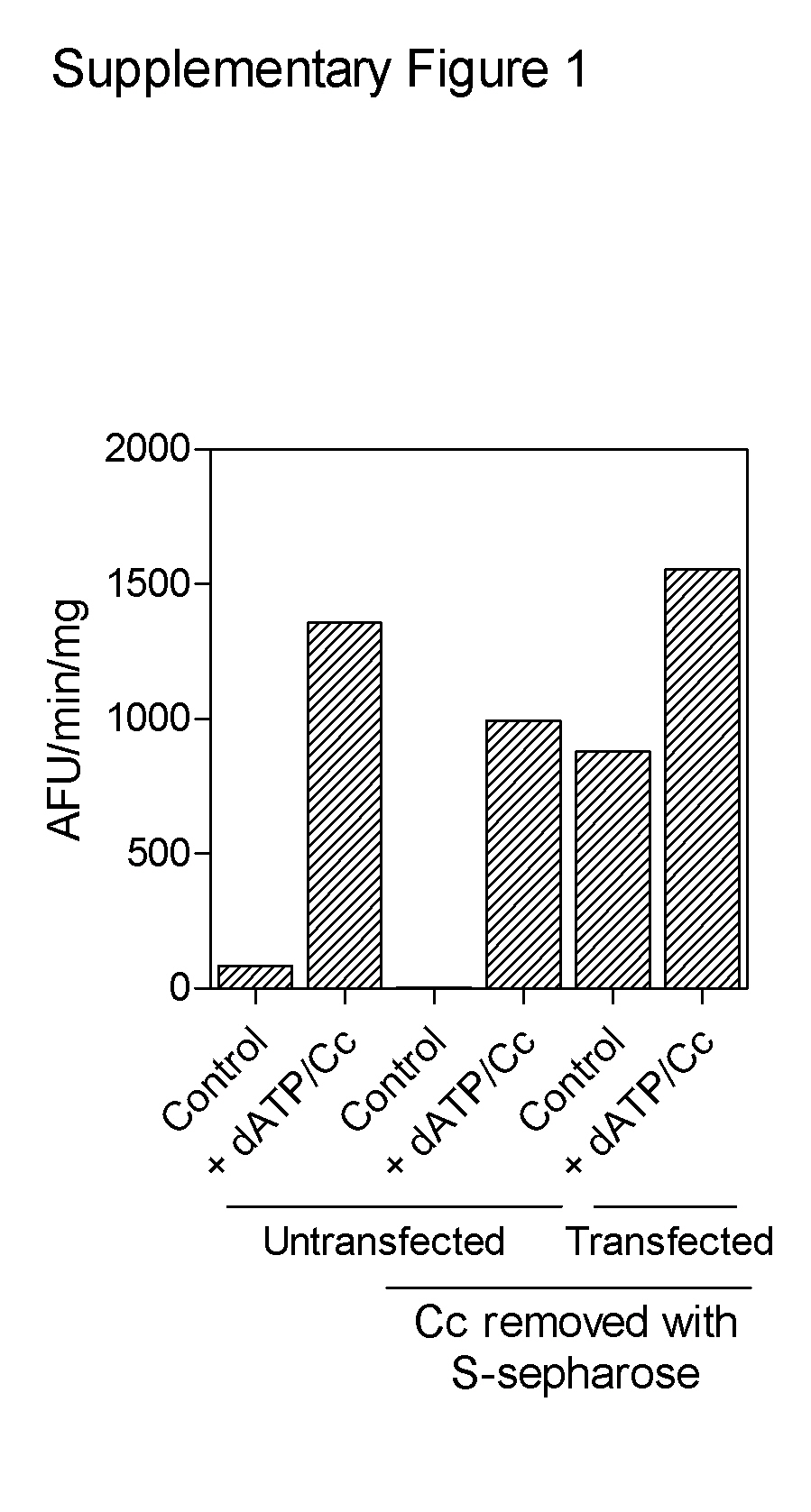

Supplement: Supplementary file 1 — Fig. S1. Depletion of cytochrome c from cell extracts. S‐100 extracts were incubated with S‐sepharose, which binds cytochrome c but not Apaf‐1, caspase‐9 or caspase‐3. Caspase‐3 activity was then assessed, either with or without addition of exogenous dATP/Cc. [file FEB4-9-1194-s001.tiff]

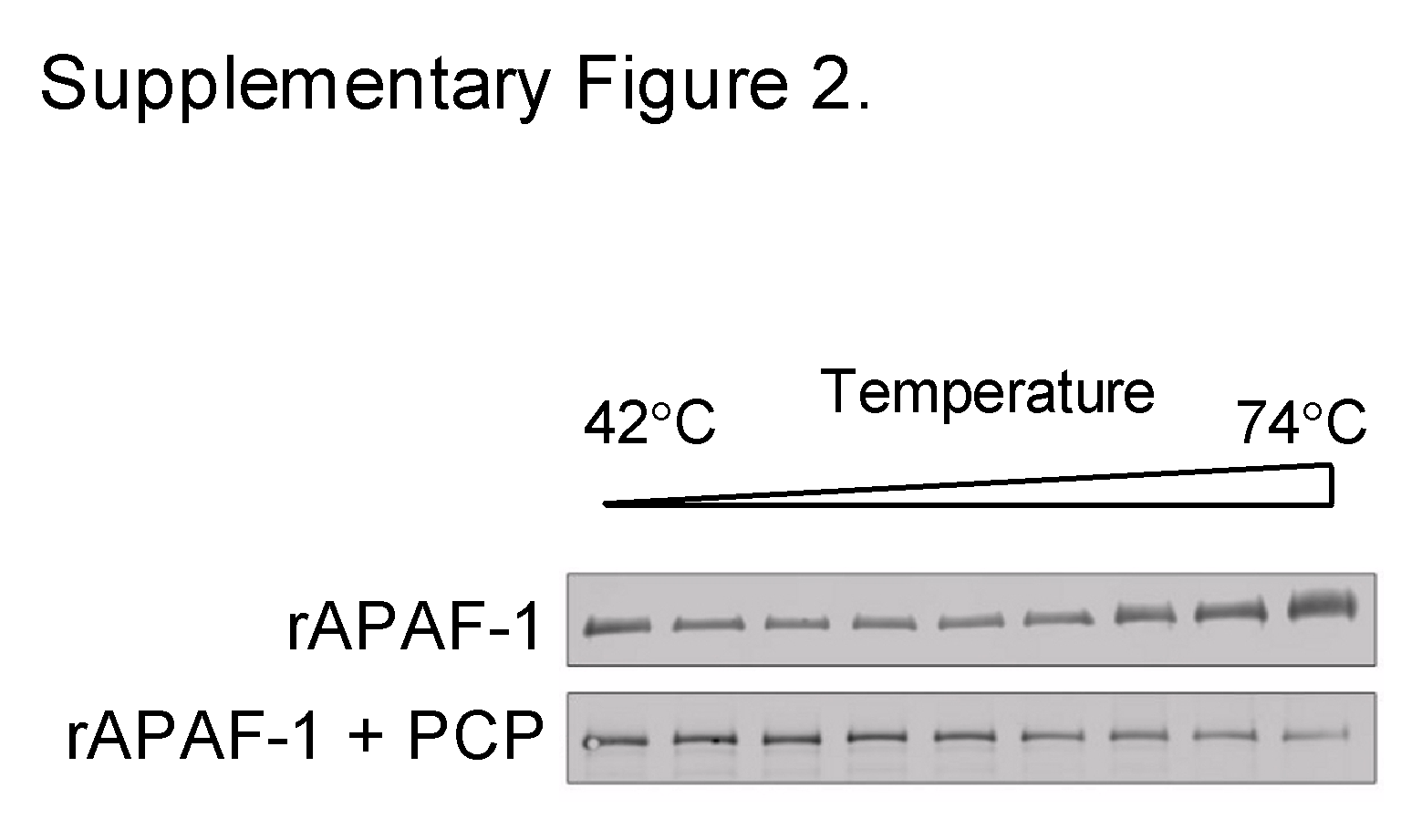

Supplement: Supplementary file 2 — Fig. S2. Thermal stability assay with Apaf‐1. Recombinant human Apaf‐1 (rApaf‐1) was expressed in Sf21 cells and purified via His‐tag affinity chromatography (see Fig. 4). Purified rApaf‐1 was then incubated with PCP (1 mm) or without (DMSO) at different temperatures to denature the protein. Denatured protein was removed by centrifugation and the amount of native rApaf‐1 was assessed by immunoblot. [file FEB4-9-1194-s002.tif]
